# Heat tolerance, thermal equilibrium and environmental management strategies for dairy cows living in intertropical regions

**DOI:** 10.3389/fvets.2022.988775

**Published:** 2022-11-16

**Authors:** Severino Guilherme Caetano Gonçalves dos Santos, Edilson Paes Saraiva, Severino Gonzaga Neto, Maria Isabelly Leite Maia, Angela M. Lees, Verassamy Sejian, Alex Sandro Campos Maia, Geovergue Rodrigues de Medeiros, Vinícius de França Carvalho Fonsêca

**Affiliations:** ^1^Department of Animal Production, National Institute of Semiarid (INSA), Campina Grande, Brazil; ^2^Research Group in Bioclimatology, Behavior and Animal Welfare (BIOET), Department of Animal Science, Federal University of Paraiba (UFPB), Areia, Brazil; ^3^Research Group in Cattle Farming (GEABOV), Department of Animal Science, Federal University of Paraiba (UFPB), Areia, Brazil; ^4^School of Agriculture and Food Sciences, Animal Science Group, The University of Queensland, Gatton, QLD, Australia; ^5^Rajiv Gandhi Institute of Veterinary Education and Research (RIVER), Kurumbapet, India; ^6^Innovation in Thermal Comfort and Animal Welfare (INOBIO-MANERA), São Paulo State University, Jaboticabal, Brazil; ^7^Brain Function Research Group, School of Physiology, University of the Witwatersrand, Johannesburg, South Africa

**Keywords:** dairy cows, thermoregulation, evolutionary adaptation, thermal equilibrium, solar radiation

## Abstract

This review makes an attempt to characterize the physical attributes of heat tolerance, thermal equilibrium and thermal stress thresholds for dairy cows living in tropical environments, with a particular emphasis on pasture-based systems. Under such circumstances, the radiant heat load is the principal climatic factor that determines rates of heat and mass exchanges between cows and the environment. This fact may explain why simple mechanistic models based on air temperature and humidity are not adequately predicting thermal stress thresholds for cattle in tropical regions. To overcome this limitation, the Index of Thermal Stress for Cows (ITSC) and Index for the time spent in shade (ITS), which account for various sources of thermal radiation, were proposed to predict autonomous and behavioral thermoregulation of cows. Overall, the evolutionary adaptation of cattle in tropics favored animals that have cutaneous surface with a skin well protected against penetration of ultraviolet solar radiation (UV), covered by a coat surface with high thermal conductivity. For Holstein breed, although predominantly black animals absorb greater levels of short-wave solar radiation, they may present better protection of skin than white ones. However, dark-colored cows in tropical pastures have potential to absorb as much as 640 W m^−2^ of thermal radiation. This amount of heat load would require close to 1,300 g *h*^−1^ of cutaneous evaporative water loss through sweating to prevent increases to body temperature, where cows do not have access to shade. Cows are motivated to reduce time spent grazing and to seek shade when solar irradiance exceeds 550 W m^−2^, levels that in equatorial latitudes are likely to occur between 08:00 and 16:00h. This information may help producers improve the welfare of cows, as they can determine more comfortable hours for them to graze, for example, by employing nocturnal grazing. Over the daytime, cows should have access to areas with shade and this could include shade provided *via* solar panels, which has the potential to improve thermal comfort and sustainability of dairy production in tropical areas.

## Introduction

Over the last century the dairy cattle populations originated from temperate climates, and that were introduced in tropical regions have acquired phenotypic characteristics that have supported thermal tolerance, *via* either natural or artificial evolutionary adaptation (1, 2). Cows in tropical environments may be exposed to higher radiant heat load than cows in temperate climates, as high levels of solar irradiance in tropics are nearly constant throughout the year, and most cows are kept in open field conditions (3–5). The most appropriate cattle phenotype for better thermal tolerance includes a set of radiative and physical properties of body surface characteristics (1, 6) that protect skin against deep penetration of ultraviolet radiation (UV) and facilitates mass and heat transference to the environment (7, 8).

The combination of a light-colored coat surface over a well-pigmented skin results more incident short-wave solar radiation being reflected, and less being transmitted throughout the skin (9). Regarding the morphological aspects of coat surface, the combination of well-settled and thick hairs favors less resistance to the diffusion of heat and water vapor throughout the boundary layer (10). While these characteristics of skin and coat surface are prominent in *Bos indicus* cattle breeds as Nellore (9), Holstein cattle and other *Bos taurus* breeds, with exception of Jersey, have skin pigmentation accompanying the coat color. In other words, predominantly white (or red) Holstein cows have a low-pigmented skin, which make them more susceptible to skin damage when exposed to high levels of short-wave solar radiation. This may explain reasons for greater abundance of predominantly black Holstein cows in these regions, as they are better protected against negative effects of UV transmission (1).

**Figure 1:**
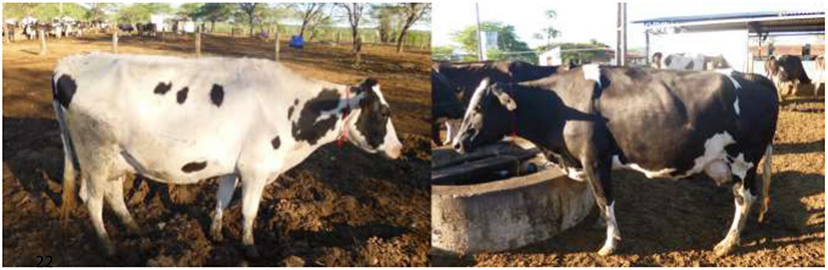
Pictures of purebred white and black Holstein cows taken in a commercial farm, Latitude = 7° S. Source: Courtesy of Severino Guilherme C.G. dos Santos.

Dark-colored cows may however absorb heat by thermal radiation twice as much as the light-colored ones (Figure 1), therefore would need to sustain greater rates of cutaneous evaporation to maintain the thermal equilibrium (11), if they are kept in tropical pastures without access to shade. The thermal radiation is the principal meteorological factor that influences thermal equilibrium of cows in tropical conditions, and consequently heat stress thresholds (12–14). This fact implies that more simplistic mechanistic models based on air temperature and humidity are very misleading to predict impacts of the thermal environment on thermoregulation and productive performance of cows in tropical areas (5, 15), as usually has been done (16). One topic of this review is to provide more appropriate approaches to improve the characterization of the thermal environment faced by a cow in a tropical environment.

Previous reviews have highlighted physiological responses of thermoregulation and productive performance outcomes of dairy cows exposed to heat stress events in temperate climates, yet few attempt to characterize the physical attributes of heat tolerance, thermal equilibrium, heat stress thresholds, and ameliorative strategies for dairy cows living in intertropical zones, particularly in Brazil, where thermal radiation is abundant and constant throughout the year. This review aims to address these topics with an emphasis on Holstein cattle. This breed was introduced in tropical areas many years ago, and currently, at least in Brazil, is the most used breed in dairy production systems. Ultimately, concerning the possible adaptive strategies to ameliorate negative impacts of solar radiation on cows, we want to highlight the Animal Agrivoltaic (17), a solution that uses photovoltaic panels to generate renewable electrical energy and to project shade for animals.

## Evolutionary adaptation of dairy cattle in Brazilian tropical regions: An emphasis on heat tolerance traits

Cattle were introduced to Brazil during the early colonization and exploration attempts; the surviving animals then reproduced and adapted to the new environment, being known as “crioulo”, “local” or naturalized (18). Zebu cattle (*Bos indicus*) were imported from India at the end of the nine-teeth century and largely disseminated over the Brazilian territory, crossing with naturalized breeds (19). With the aim at improving dairy productivity, during the 1920s and 1930s, there were attempts to introduce several European cattle breeds as Holstein, Jersey, and Brown Swiss (20). Although more productive, these animals were not adaptable to the new environment, especially to the thermal conditions (2).

Around the 1940s Brazilian farmers began to cross the Gir (an imported *Bos indicus* breed) with the Holstein (20–22), in order to attain the hybrid vigor, and to develop a high producing yet heat-tolerant breed. Indeed, milk yield of Holstein x Gir crosses averaged 2,574 kg per 305 days, significantly higher than the 1,600 kg produced by Gir in India (23, 24); this superiority increases with the Holstein contribution (e.g., from 1/2 to 7/8) (22). Most dairy cattle population in Brazil is currently represented by crossbreds (mainly Holstein x Gir), mostly kept under pasture based-systems (21). Furthermore, as a result of almost 100 years of evolution in tropical regions, the phenotypic aspects of purebred Holstein cattle are much different when compared with contemporary Holsteins living in temperate climates, particularly that associated with heat tolerance (8–10). For instance, the effective thermal conductivity (W m^−2^ K^−1^) of body surface of Holstein cows living in Brazil was reported to be much higher than those in temperate conditions, which is explained due to the lower coat thickness of the Brazilian cows (2.4 vs. 25 mm) (1).

**Figure 2:**
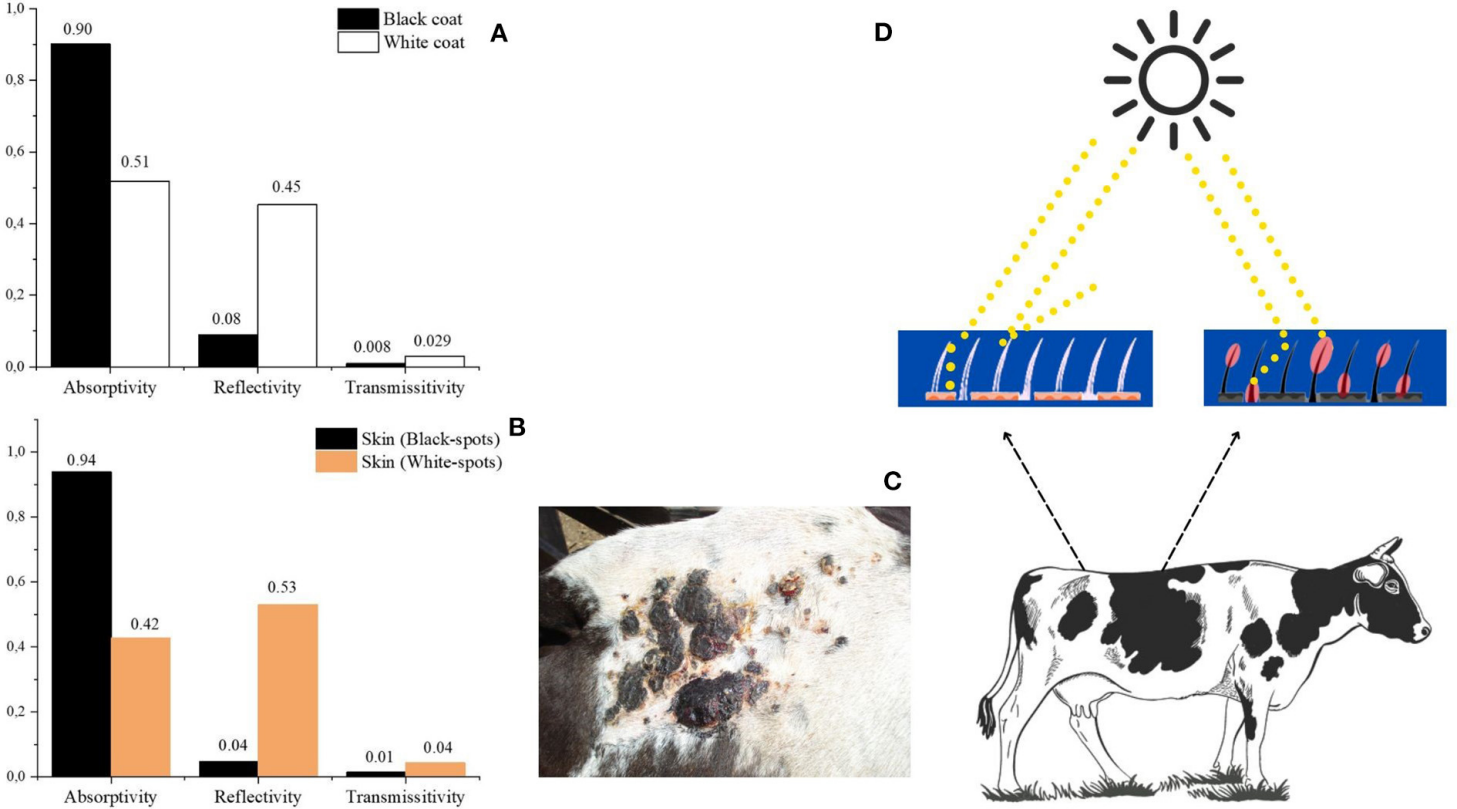
Radiative properties of the coat **(A)** and skin **(B)**; skin burn **(C)** in the white spot of Holstein cow managed in open pasture of a tropical region (Source: Courtesy by Roberto G. da Silva). Measurements of the radiative properties were performed with a radiometer [DaSilva et al. (6)]. **(D)** Dotted yellow lines represents the electromagnetic photons of energy that can be reflected, transmitted and absorbed (red circles) as heat over the coat and skin surface.

Heat tolerance is determined by the relationship between metabolic heat production and the ability to dissipate body heat (2). The coat color is the primary determinant of the amount of radiant heat absorbed over the body surface, sourced from short-wave solar radiation (i.e., spectral range from 300 to 3,600 nm). Although black-haired cows absorb more incident radiation (*circa* 91%) than the white-haired ones (close to 35%) ((9), Figure 2), Holstein cattle population in Brazil is mostly represented by black animals (6, 8, 9, 25, 26, 60). This information seems to evidence that adaptation of Holstein cattle in tropical regions favored selection of traits that are of greater importance to animal fitness than a body surface that absorbs less radiant heat.

Light coats of European cattle breeds, with exception of Jersey, coexists with a low-pigmented skin (9, 10) in response to a gene action that blocks melanogenesis (27). Breeds or animals with this characteristic are likely to be more susceptible to the harmful effects, e.g., skin burns and neoplasia (Figure 2), of the ultraviolet radiation (spectral range between 200 and 390 nm). Mixed colored Holstein cows in equatorial regions of Brazil were reported with serious cutaneous tissue damage only in white spots (25). Because black spots are over a well pigmented skin, less UV radiation is transmitted throughout dermis and epidermis (Figure 2).

Black cows however present higher absorption of thermal radiation and therefore have more body heat to be dissipated than white-haired cows, particularly if they are exposed to solar radiation. The thermal balance of the body surface is determined by a combination of radiative properties and arrangement of individual hairs, which in turn, is a function of density and inclination angle of the hairs at the skin surface (28). A body surface with a less-dense coat, thick, short, and well settled hairs characterize most cattle breeds well adapted to tropical conditions, including Holstein (7). These characteristics favor less still air to be trapped within the boundary layer, which in turn, gives less resistance to the diffusion of heat and water vapor, and enhances dissipation of heat and mass (mainly through sweating) to the environment (1).

The set of black and well-pigmented skin (*h* = 0.75), hair diameter (*h* = 0.63), sweating rate (*h* = 0.43), age at first calving (*h* = 0.23), and milk yield (*h* = 0.34) were found to have relatively high values of heritability, and favorable genetic correlations between them (29–31). Breeding programs therefore can select more heat tolerant, but still productive Holstein and Jersey cows to be raised in open field conditions of tropical regions. Moreover, with advances of genomic tools, it was possible to identify a specific gene that confers animals with a short and sleek hair coat, the SLICK haplotype (*slick hair*), which allows faster selection in order to enhance thermal conductivity of the body surface, and heat tolerance of cows. Holsteins cows with slick hair gene had superior thermoregulatory ability and lesser decay in milk yield during the summer, when compared to wild-type Holstein cows that did not have the slick hair gene (32). Further discussion about other possible genes that confer better thermal tolerance of dairy cattle can be found at Silpa et al. (33).

## Thermal equilibrium of cows in tropical areas: An emphasis on thermal radiant environment

Living organisms are physical systems in which thermal energy is produced continuously by means of metabolic processes and exchanged (gains and losses) with the surrounding environment (34). The thermal equilibrium of a cow can be described as:


(1) $$M\;+\;R_{C}\alpha +\;S\;\pm \;R_{L}\pm \;C_{S}\pm \;K\;-\;C_{R}-\;E_{S}-\;E_{R}=0$$

Where ***M*** represents the metabolic heat production; for lactating Holstein and Jersey cows (Average milk yield of 25 kg) in tropical regions the metabolic heat production was reported to be between 180 and 230 W m^−2^ (34, 35). ***R***_***C***_ is the heat absorbed by short-wave solar radiation, which depends of the absorptance of the coat surface (**α**); ***R***_***L***_ is the thermal energy exchanged by long-wave radiation; ***S*** is the rate of thermal energy storage; ***C***_***S***_ is the heat exchanged by surface convection; ***C***_***R***_ is the heat transferred by respiratory convection; ***E***_***S***_ is the heat transferred by cutaneous evaporation; and ***E***_***R***_ is the heat transferred by respiratory evaporation.

**Figure 3:**
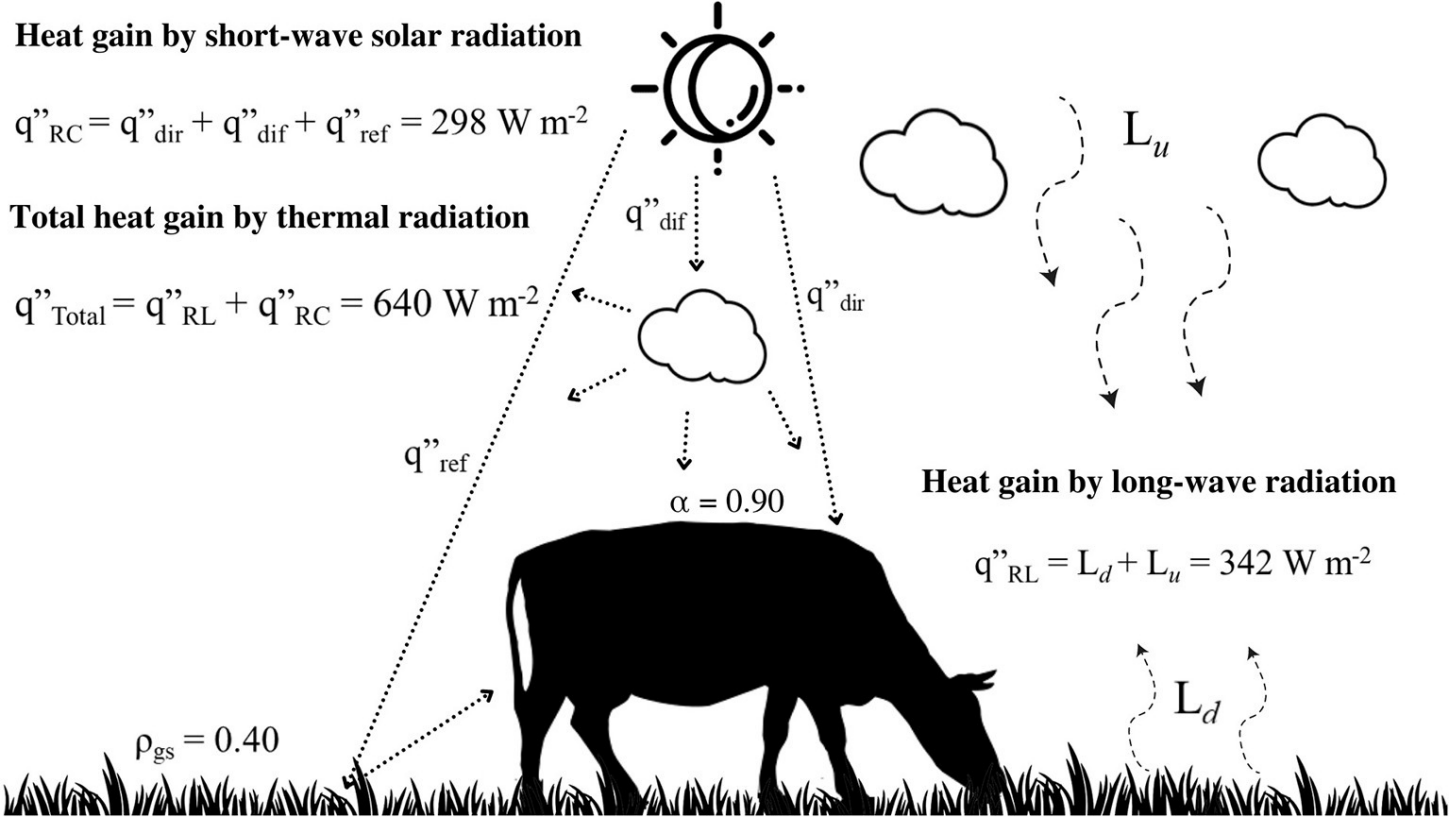
Representation of the thermal radiation absorbed by a dairy cow in a tropical pasture, at 12:00h, January, 20, Lat. 5° S (DaSilva et al. (14)). The ρ_gs_ is the reflectance coefficient of a green grass, and α is the absorptance coefficient of a black coat surface. The rates of total long-wave radiation (*q*”_*RL*_, W m^−2^) are the sum of downward radiation, emitted from the sky (*L*_*u*_), and upward radiation emitted from the ground surface (*L*_*d*_). The rates of heat absorbed by short-wave solar radiation (*q*”_*RC*_) encompass direct (*q*”_dir_), diffuse (*q*”_dif_), and reflected (*q*”_ref_) solar radiation.

The sensible heat transfer encompasses conductive (***K***), convective (***C***_***R***_ and ***C***_***S***_) and radiative heat transfer (***R***_***C***_ and ***R***_***L***_, Figure 3), while the evaporative heat transfer encompasses cutaneous (***E***_***S***_) and respiratory (***E***_***R***_) evaporation. The direction and rate of heat transfer by each of the three sensible routes depend on a temperature difference between the surface of the animal and that of the environment. However, the temperature of the surrounding air is not the most relevant meteorological variable for determining rates of sensible heat exchanges by a cow (at standing position) in a tropical pasture (16). In this case, sensible heat exchanges, particularly by long-wave radiation, are totally dependent of a mean radiant temperature, an environmental temperature that accounts all sources of thermal radiation emitted from the surrounding environment, i.e., ground-surface, objects, trees and sky (14).

Holstein and Jersey cows kept indoor partially offset the metabolic heat production by means of surface convection and long-wave radiation when the temperature of the surrounding air ranges between 15 and 25°C. Under such circumstances, evaporative heat transfer, mainly through cutaneous surface may account for only 20–30% of the metabolic heat dissipated to the environment. On the other hand, when the air temperature is above 28°C, the rates of cutaneous evaporative water loss become the principal route of heat elimination, by accounting for 80% of the total metabolic heat production (35, 36). Indeed, the upper critical temperature for dairy cows is predicted to be within 25 and 28°C, depending on its levels of metabolic heat production (37). This temperature threshold is however not relevant for cows outdoors, especially if they are exposed to high levels of thermal radiation. Recent research shows that Holstein cows kept outdoor in a tropical environment, and experiencing same level of air temperature of their counterparts kept indoor, increased by 50% the rates of cutaneous evaporative water loss, were more vasodilated, and stored more body heat (Fonsêca et al.; in preparation).

This result emphasizes thermal radiation as the principal meteorological component at determining thermal balance of cows in tropical environment. A black-haired Holstein cow in a pasture have potential to absorb as much as 640 W m^−2^ of thermal radiation during the hottest hours of the day, e.g., from 09:00 to 16:00h (14; Figure 3). The short-wave solar radiation may account to 298 W m^−2^ or 47 % of the total thermal radiation absorbed, while long-wave radiation (342 W m^−2^), most of which emitted from the ground surface, accounts to 53%. This amount of heat load can represent threefold the metabolic heat produced by a cow (~ 200 W m^−2^), which in turn, would need to dissipate close to 850 W m^−2^ in order to maintain their thermal equilibrium. For doing so, cows would need to produce and to evaporate up to 260 g *h* m^−2^ or 1,300 g *h*^−1^ of sweat (Surface area of a cow, m^2^ = ~ 5).

**Figure 4:**
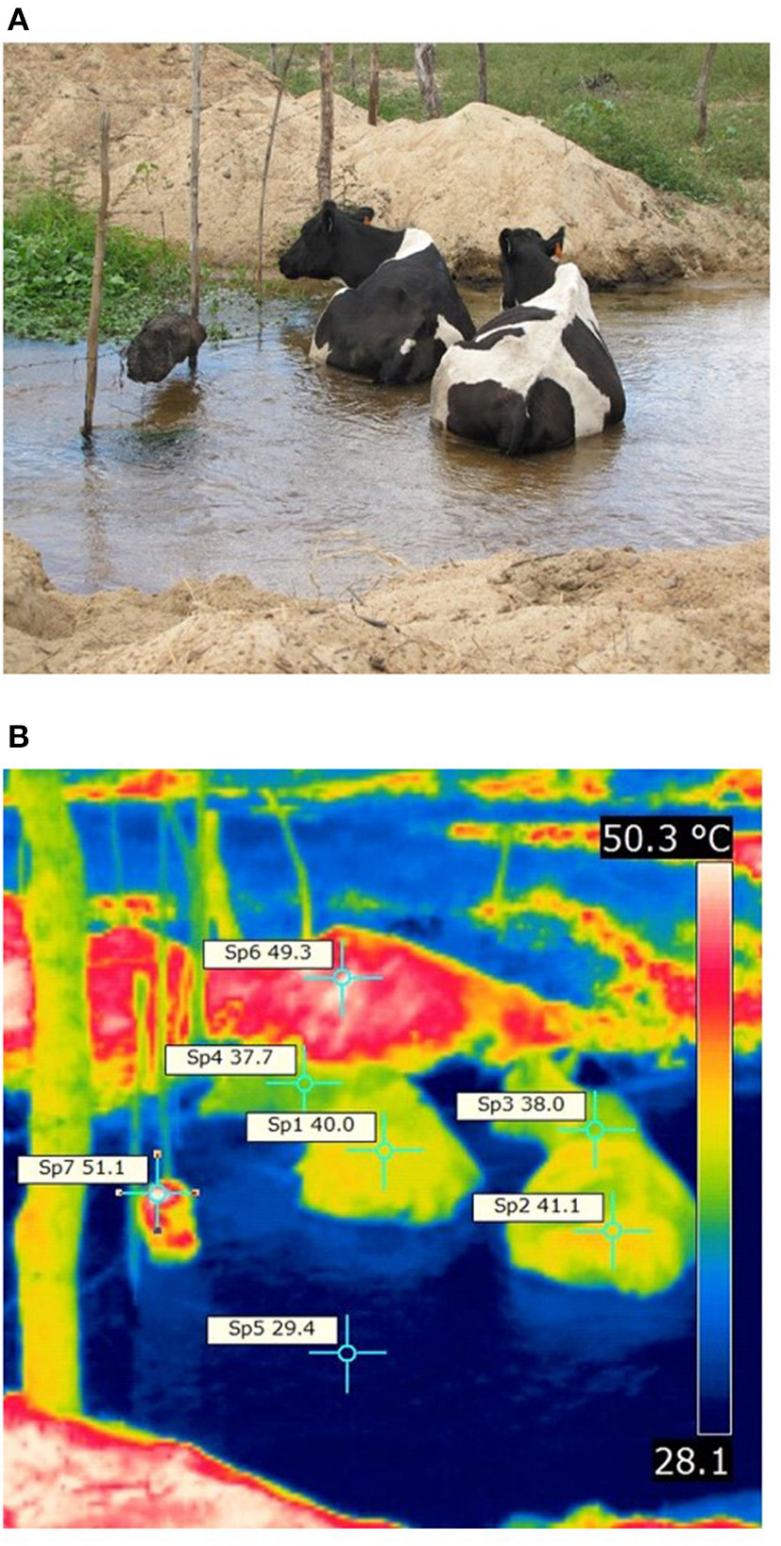
Holstein cows employing conductive and convective cooling with the water river **(A)**. Thermogram taken in an equatorial latitude **(B)**. Source: Courtesy by Alex S. C. Maia, innovation group of thermal comfort and animal welfare (INOBIO-MANERA). Dotted yellow lines (D) represents the electromagnetic photons of energy that can be reflected, transmitted and absorbed (red circles) as heat over the coat and skin surface.

By adjusting behavior, cows however can manipulate rates of heat exchanges with the surrounding environment; for example, by seeking shade, they can avoid absorption of high amount of radiant heat load, thus decreasing requirements for evaporative water loss through cutaneous surface. Moreover, by lying down in a shaded surface, cows may be able to dissipated body heat by conduction depending on ground surface temperature. The critical level of short-wave solar radiation that motivates cows to stop grazing and to seek shade is between 500 and 700 W m^−2^ (38). In the absence of shading resources, cows can also employ conductive and convective cooling by lying down in sources of water (12, 13; Figure 4). It is possible to see that the two cows in the Figure 4 can lose body heat due to a positive thermal gradient between body and water surfaces. However, this behavior can be critical in terms of dissemination of diseases as mastitis, especially soon after the post dipping procedures.

## Predicting heat stress responses of dairy cows living in tropical regions

Several indexes have been extensively used to predict the environmental thermal comfort threshold for cattle, such as the temperature-humidity index, THI (39); effective temperature, ET (40); black globe humidity index, BGHI (41); equivalent temperature index, ETI (42), heat load index, HLI (43). Overall, some of these indices were based on animal responses under temperate region, and in controlled climatic chambers, which in turn do not well represent complexity of the thermal radiant environment faced by dairy cows in tropical regions (34). The effectiveness of five indices against thermoregulatory responses of Holstein cows kept in open field conditions of an equatorial region of Brazil were tested (5). Unsurprisingly, results confirmed that THI and BGHI were not correlated with rectal and respiratory rate of cows. On the other hand, the Heat Load Index [HLI; (44)] presented significant correlations with thermal responses of cows, a single variable who accounts effects of air temperature, humidity, wind speed and solar irradiance, which was initially developed for feedlot cattle in Australia.

Although the importance of air temperature and humidity for the heat exchange processes between animals and environment, thermal radiation assumes greater importance for cows kept in tropical pastures. A Thermal Stress Index for Cows (ITSC) was proposed by DaSilva et al. (5) based on meteorological measurements in an equatorial region, in addition to physiological responses of 814 Holstein cows. By using a principal component analyses the physiological variables respiratory rate (*R*_*R*_, breath min^−1^), rectal temperature (*T*_*R*_, °C), skin temperature (*T*_Skin_, °C), body surface temperature (*T*_*S*_, °C), cutaneous (*E*_*C*_, W m^−2^) and respiratory (*E*_*R*_, W m^−2^) water loss of cows were summarized into a single variable, the ITSC. The ITSC was then fitted against air temperature (*T*_*A*_, °C), wind speed (*U*, m s^−1^), humidity (*P*_*V*_,kPa), and effective radiant heat load (ERHL, W m^−2^) (a meteorological variable who accounts effects of short-wave and long-wave radiation), by employing multiple regression analyses. The best fitted equation chosen based on higher *R*^2^ in order to determine values of ITSC was:


$$\begin{array}{l} \text{ITSC}=\text{77}.\text{1747}+\text{4}.\text{8327}{\text{T}}_{\text{A}}-\text{34}.\text{8189 U}+\text{1}.\text{111}{\text{U}}^{\text{2}}\\ \;+\text{118}.\text{6981}{\text{P}}_{\text{V}}-\text{14}.\text{7956}{{\text{P}}_{\text{V}}}^{\text{2}}-\;0.\text{1}0\text{59 ERHL} \end{array}$$

**Figure 5:**
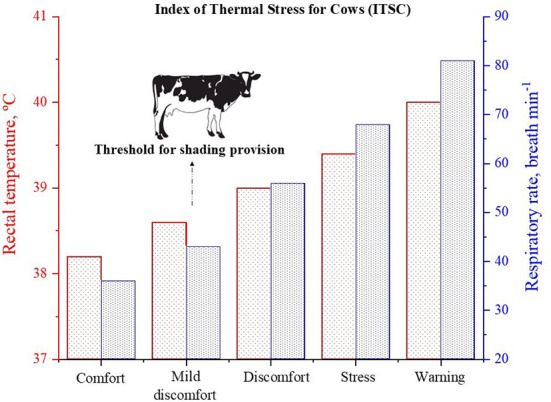
Mean of rectal temperature and respiratory rate of Holstein cows (*n* = 840), according to the heat stress thresholds of the Index of Thermal Stress for Cows (ITSC). Mean values were sourced from DaSilva et al. (5). Comfort (ITSC ≤ 150), Mild discomfort (151 < ITSC ≤ 200), Discomfort (200 < ITSC ≤ 250), Stress (250 < ITSC ≤ 350), Warning (ITSC > 351).

The values obtained for ITSC were fitted against physiological responses of cows, and significant correlations were observed with rectal temperature (*r* = 0.472), respiratory rate (*r* = 0.793), skin surface temperature (*r* = 0.755) and sweating rate (*r* = 0.570). These correlations derived five heat stress thresholds for cows (Figure 5). For instance, when value of ITSC is greater than 200, cows must be provided with access to shade. Even for cows managed in free-stalls under semi-intensive production systems, with occasional or free access to external paddocks, values of ITSC may indicate when they should be housed again. Complementary to the ITSC, another thermal stress index was proposed to predict the time spent in the shade by dairy cows in tropical areas, the shade time index (ITS) (45). The ITS can be calculated based on local values obtained for air temperature (*T*_*A*_, °C), relative humidity (*R*_*H*_, %), solar irradiance (*S*_*R*_, W m^−2^), and black-globe temperature in the shade (*T*_*G*_, °C), as follow: IST = – 14.32 (0.79*T*_*A*_) – (0.56*R*_*H*_) + (0.041*S*_*R*_) + (1.58*T*_*G*_). In addition to the ITSC, the IST can be employed in order to improve daily management of cows kept under pasture based-systems, for example, by determining more comfortable hours for them to graze.

## Heat abatement strategies for dairy cows in tropical environment: An emphasis on shade use

Shade is any region of space not traversed by a radiation beam (34). Benefits coming from shade use by animals are due to the abatement of radiant heat gain through two main sources. First, from direct and diffuse short-wave solar radiation, where on hottest times, by seeking shade, animals could avoid levels of impinging solar irradiance as much as 1,200 W m^−2^. Second, animals in shade may also have decrease radiant heat gain due to less long-wave radiation emitted from a shaded surface, which in turn, is directly proportional to its absolute temperature. Ultimately, animals could also avoid heat gain by conduction when lying down on cooler shaded surfaces. The provision of shade (natural or artificial) is therefore of particular importance at alleviating heat stress of cows kept under pasture based-systems of tropical areas. In particular, by taking into account shade availability and level of solar blockage provided by the shade structure (46–48).

A cow should be provided with minimum of 3 m^2^ of shaded area, otherwise benefits of its use is likely to be minor, thus reducing cow's motivation to keep in the shade (34, 49). High density of animals in the shade may impair air displacement, thus decreasing efficiency of the convective evaporation over the body surface of animals. Studies have shown that cows with access to 9.6 m^2^ of shade/cow spent twice as much time in shade compared to the availability of only 2.4 m^2^ shade/cow, had lower panting scores and expressed less agonistic behaviors (50, 51). A cow also should be provided with a shade structure that blocks as much as possible the direct and diffuse solar radiation (52). Indeed, they spent greater time within the shade projected from structures that blocked 100% of solar radiation against shade-cloth structures that blocked 50 and 70% (45).

**Figure 6:**
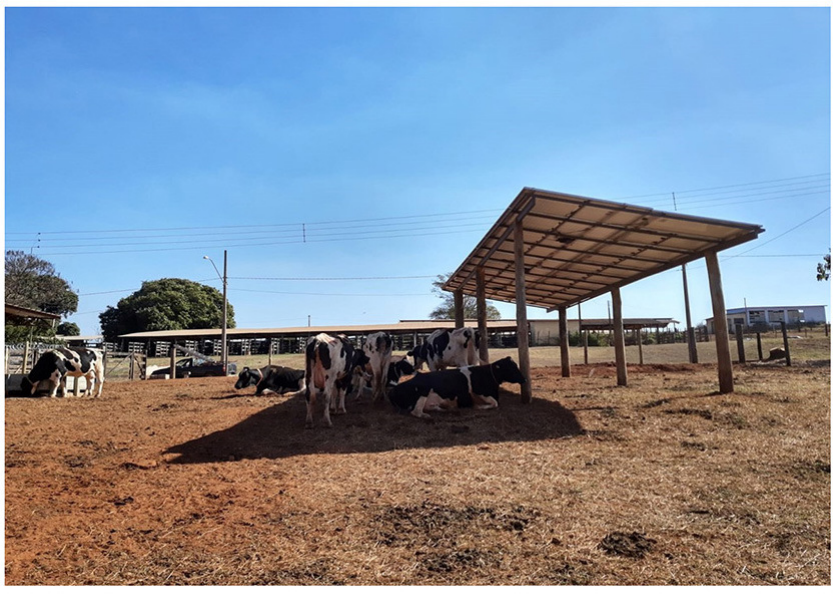
Holstein cows using shade projected from photovoltaic panels Latitude = 21° S (Source: Courtesy of Alex S. C. Maia, Innovation Group of Thermal Comfort and Animal Welfare (INOBIO- MANERA).

One good option is a shade provided by photovoltaic panels, by using the concept of “Animal Agrivoltaic”, recently developed by the Innovation Group of Thermal Comfort and Animal Welfare [Inobio-Manera; (17)]. The Animal Agrivoltaic is a type of co-generation system that provides high quality shade for animals, production of sustainable animal protein, and generation of renewable electrical energy in the same area. Cows in the shade projected by photovoltaic panels can experience nearly by 100 W m^−2^ less radiant heat load than if they were in a shade projected by shade-cloth structure that blocks 90% of solar radiation (Figure 6). The possibility of such integrated systems cast the interest of policy makers and governmental agencies in Brazil and around the world, as a potential solution to offset carbon emissions derived from livestock production systems (17, 53, 54). Another strong motivation for the implementation of sustainable co-generation systems using photovoltaic panels is the continuous decrease in the price of photovoltaic panels (from US$ 3.90 *Wp* in 2006 to US$ 0.39 *Wp* in 2016; 5% expected annual price drop; (55)) as well as the development of new technologies expected to increase the efficiency of energy conversion (from 18 to 45% using Single-Junction GaAs, Thin-Film Crystal).

Even in the shade cows may experience levels of air temperature higher than its body surface, a condition that makes heat to be absorbed by the animal from the surrounding environment. Under such circumstances, spraying water over the skin can enhance mass and heat transfer through the cutaneous surface, thus avoiding body heat storage and thermal stress (56, 57). Although several studies have been showed benefits of sprinklers on thermoregulation and performance of dairy cows kept in housed conditions (58, 59), applicable solutions are needed to be proposed for cows managed in open pastures. For instance, shading and water spraying cows with a simple hand-held pump every 1 *h*, from 10:00 to 15:00, decreased body heat storage, requirements for evaporative water loss through panting, and increased milk yield by 3.0 kg day^−1^ cow^−1^. This type of solution can be practical in small dairy farms (57). However, further studies should determine optimum amount of water that must be sprayed in order to offset requirements of cows for evaporative cooling mechanisms through sweating and panting.

## Final remarks

This review highlighted the significance of the thermal radiant environment as the principal meteorological stressor for dairy cows living in intertropical regions, and discussed about more appropriate mechanistic models in order to predict heat stress thresholds for them. A coat surface with high thermal conductivity over a well-pigmented skin is the best phenotype for cows kept in open pasture, and for Holstein, a combination that only occurs in black-haired cows. As black cows receive large amount of thermal radiation sourced from short-wave and long-wave radiation, the provision of shade is then mandatory, particularly when solar irradiance exceeds 550 W m^−2^, levels that motivate cows to stop grazing. Ultimately, shade could be provided by solar panels, as they can efficiently buffer the radiant heat load on cows, generate clean and renewable electrical energy, thus improving sustainability of dairy cow production in tropical environment.

## Author contributions

Conceptualization, methodology, and writing original draft—manuscript: SS and VF. Writing original draft—figures: VF, SS, MM, and AM. Writing—review and editing: VF, ES, AL, VS, AM, GM, and SG. Visualization: VF and AL. Supervision: ES and VF. Funding acquisition: ES and SG. All authors contributed to the article and approved the submitted version.

## Conflict of interest

The authors declare that the research was conducted in the absence of any commercial or financial relationships that could be construed as a potential conflict of interest.

## Publisher's note

All claims expressed in this article are solely those of the authors and do not necessarily represent those of their affiliated organizations, or those of the publisher, the editors and the reviewers. Any product that may be evaluated in this article, or claim that may be made by its manufacturer, is not guaranteed or endorsed by the publisher.
